# Jealousy: A comparison of monogamous and consensually non-monogamous women’s experience

**DOI:** 10.1080/28324765.2023.2283006

**Published:** 2024-01-16

**Authors:** Joli Hamilton, Nicholas R. Morrison, Ayla N. Gioia

**Affiliations:** a Department of Psychology, Westfield State University, Westfield, MA, USA; b Department of Psychology, Hofstra University, Hempstead, NY, USA

**Keywords:** jealousy, monogamy, consensual non-monogamy, interpretative phenomenological analysis

## Abstract

Jealousy, the emotional experience of feeling threatened when a valued relationship is interrupted by an outside party, is often described as a subjectively negative experience. Research suggests that jealousy is a source of conflict in monogamous relationships, yet little empirical research has examined the experience of jealousy in consensually non-monogamous (CNM) relationships. Given the differences that exist between monogamous and CNM relationships (e.g. differences in prioritization of autonomy and agency) and the scant empirical literature regarding consensual non-monogamy, it is important to further examine the role of jealousy in different relationship structures. The current study aims to explore the lived experiences of jealousy in cis-women across these relationship structures. The authors selected interpretative phenomenological analysis (IPA), a qualitative approach known for its robustness in supporting in-depth investigations of idiographic psychological phenomena, to explore experiences of jealousy in both monogamous (*n* = 6) and CNM (*n* = 5) cis-gender women. Results suggest the way participants made sense of jealousy was influenced by the relationship structure they participated in. IPA revealed four experiential themes: “jealousy’s somatic sensations”, “jealousy’s accompanying emotions”, “norms and beliefs about jealousy”, and “strategies for managing jealousy”, each of which was organized into subthemes. Implications for relationship structures and clinical practice are discussed.

Across the extant literature, jealousy is described as negative and subjectively unpleasant, yet it is ubiquitous throughout known cultures and various populations (Chung & Harris, 2018). Many go to great lengths to “cure”, reduce, or avoid jealousy completely (Leahy, 2018). Often jealousy is socially scorned as a character flaw largely attributable to low self-esteem or lack of relational skills (Baumgart, 1990; de Silva, 2004). Yet, other challenging emotions, such as sadness, fear, and anger, are conceptualized as protective and valuable in addition to their uncomfortable features. When an emotion is categorically defined by its negative nature, there is a risk that emotion will also carry a connotation of shamefulness, particularly in the context of relationships.

Jealousy has been a source of relational trouble and dramatic fascination for thousands of years. From ancient Greek mythology to modern reality television, triangles of jealousy spark the imagination and remind us that any time we have a valuable relationship, there is the potential for that relationship to be interrupted by another person. Jealousy is largely described in the literature as a negative and unwanted, though a smaller proportion of the research points to jealousy as a potentially valuable emotional experience (Pfeiffer & Wong, 1989).

Put most simply, jealousy is an emotional experience felt when one detects a threat to a valued relationship. Jealousy is a social emotion that occurs in a relational triangle made of the jealous one, the valued other, and the perceived interrupter when the jealous one feels a threat to the relationship between themselves and the valued other (see Figure 1; Hamilton, 2019; White & Mullen, 1989). The perceived interrupter may be an identifiable “real” person who is seen as a threat to the relationship, or the interrupter may be imagined by the jealous one. The relational triangle is a key element in differentiating between jealousy and envy, which occurs in a dyad when the envying person desires what the other person has or is.

**Figure 1.**
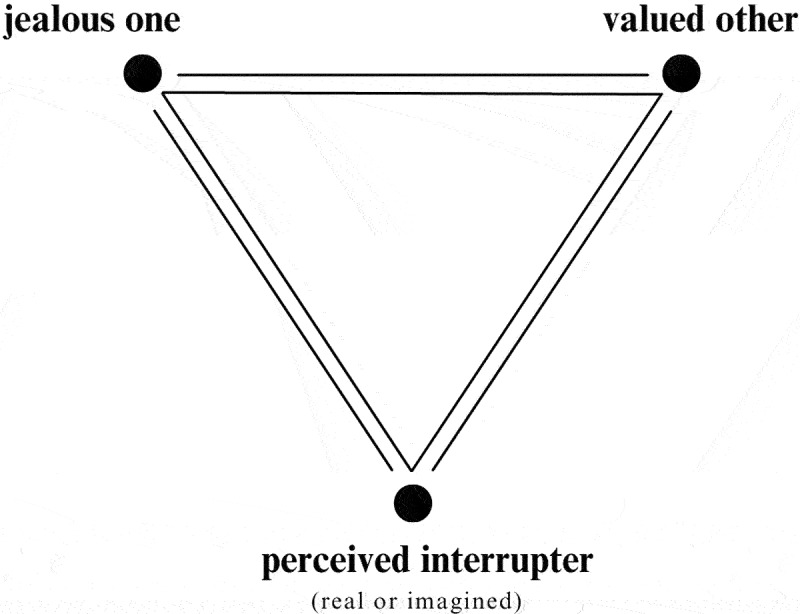
The jealousy triangle.

## Monogamy and consensual non-monogamy

Historically, the default position of research into relational styles has centered on monogamy (Conley et al., 2013). Since monogamy is a situation of exclusive pair bonding to one singular valued other, typically called “a couple”, the potential for jealousy appears any time a third party enters the relational imagination (Rydell et al., 2004; Salovey, 1991). Some research points to the evolutionary advantages of jealousy as a progenially protective strategy (Buss, 2000), while others emphasize the advantageous protectiveness of attachment-caregiving (Barelds et al., 2017; Chung & Harris, 2018). However, across the research literature consensual non-monogamy (CNM) is rarely considered as more than an anomaly amongst the “naturalness” of monogamous relationships (Buss, 2013; Fisher, 2016; Hamilton, 2019).

CNM is the practice of having or being open to multiple intimate relationships simultaneously (Conley et al., 2017). Individuals may use the term CNM to denote a variety of non-monogamous relationship structures and norms. There is not yet a universally accepted definition, though the various definitions typically focus on honesty and informed consent practices (Conley et al., 2013; Haupert et al., 2017; Moors et al., 2021). It is important to differentiate between CNM, which is consensually negotiated between partners and “cheating”, which is non-consensual infidelity in a relationship.

Forms of CNM have existed across cultures and throughout history according to anthropological data (Emens, 2004). Recent studies have shown that approximately 4–5% of the US population is actively participating in CNM and up to 20% have explored it at some point in their lives (Moors et al., 2021). Polling data suggests that even larger percentages of younger generations consider CNM their ideal relationship structure (YouGov, 2020).

While it is typically understood that monogamous individuals will prioritize relational moves that secure and reinforce intimate exclusivity (Fisher, 2016), consensually non-monogamous (CNM) individuals prioritize individual autonomy and agency, as well as expansive relationship building beyond the dyadic style of monogamous pairing (Balzarini et al., 2019). Additionally, CNM individuals intentionally create the opportunity for multiple valued others; thus, there is a strong possibility that the situation of a social triangle will form naturally over the course of non-monogamous life (Haupert et al., 2017).

Although there has been a recent uptick in both general media and academic research efforts surrounding CNM, extant research suggests we are far from having sufficiently explored CNM relationship structures (Scoats & Campbell, 2022). Notably, in a systematic review of research on attitudes toward CNM among emerging adults, Sizemore and Olmstead (2017) identified multiple ongoing shortcomings in the field when studying this population, including limitations related to overall methodology, focus, and sampling characteristics. The authors noted that no studies were found that solely used qualitative methods and called for future research using these methods to study the attitudes of CNM. Additionally, participants across research studies tend to be predominantly White and heterosexual. The shortcomings of CNM research are unsurprising given the stigma and social status of this marginalized population, and become further exacerbated when considering the underrepresented experiences of individuals from additional minority-status groups (e.g., CNM individuals who identify as trans, of color, disabled/differently abled, etc.). These considerations underscore the importance of better understanding the lived experiences of these individuals. Given the evidence that CNM relationships can be at least as satisfying as monogamous relationships (Balzarini et al., 2019; Rubel & Bogaert, 2015), additional calls for research have emphasized exploring more nuanced questions about the experience of CNM, including the role of jealousy (Balzarini & Muise, 2020).

## Applications in mental health

Jealousy can be a significant problem in mental health, leading to a large collection of emotional and safety issues that interfere with personal and relational emotional functioning. For example, cognitive behavioral therapy (CBT) models of jealousy differentiate between jealousy as an emotion and the relational strategies a person might utilize to increase relational stability or satisfaction (Leahy & Tirch, 2008). Acute jealousy identified in the clinical setting can be the cause of suffering on the part of individuals and partnered pairs, and can even be a causal factor in violence and homicide (Buss & Abrams, 2017).

A CBT perspective positions jealousy as a valuable protective mechanism in romantic relationships (Buss & Abrams, 2017; Leahy & Tirch, 2008). However, current research is grounded in a mono-normative perspective that is centering monogamous relationship standards, potentially creating a problematic bias that can interfere with applications of common treatment strategies in situations of non-monogamous relational experiences of jealousy (Witherspoon, 2018). This suggests there can be different therapeutic approaches for different relationship structures, yet the default treatment approach in clinical/counseling settings is based on a monogamous paradigm. To the extent that this default is used, the non-monogamous viewpoint is excluded, and the quality of care could be undermined in CNM populations (Herbitter et al., 2021. In addition, individuals in relationship structures such as CNM or polyamory can face obstacles to quality mental healthcare. This is due in part to the stigma and stereotyping frequently experienced by such individuals (APA, 2021; Conley et al., 2013; Rubel & Bogaert, 2015). These difficulties can be further exacerbated by clinicians’ biases and lack of expertise or even competence in navigating these issues with their clients (Finn et al., 2012; Katz & Graham, 2020; van Tol, 2017).

Several recent studies have called for increased information and training for mental health care providers serving marginalized sexualities populations (Herbitter et al., 2021). It is important to address the mental healthcare issues of non-monogamous individuals and families for multiple reasons. From the mental healthcare professional’s standpoint, working with clients in unfamiliar relationship structures, such as CNM, could cause undue job stressors. Researchers have previously commented on the lack of adequate training about diverse relationships and marginalized sexualities in clinical training programs (Herbitter et al., 2021). Additionally, mononormativity, the privileging of monogamy, may impact clinicians’ self-awareness in imposing monogamous standards that may be inappropriate in the treatment of CNM clients (Cassidy & Wong, 2018; Graham, 2014). From the client’s perspective, the complex stigma associated with non-monogamy in the clinical setting itself as well as in legal, professional, and social contexts (Mahar et al., 2022; Witherspoon & Theodore, 2021) will likely lead to greater mental health needs. These increased mental health needs would of course be a burden to the client and also put increased pressure on the mental healthcare system. Thus, a greater understanding of phenomenological perspectives of jealousy will yield various benefits including the opportunity to consider what might make for more effective and targeted counseling interventions across relationship structures.

## Present study

Given the default understanding of jealousy in society from a monogamous paradigm, coupled with the historical consistency and increasing interest in CNM, consideration of jealousy from both monogamous and non-monogamous perspectives seems to be a necessary elaboration upon the existing literature on jealousy, including quantitative work that has examined similarities and differences in this area (e.g., Mogilski et al., 2019). Also, an enhanced understanding of both monogamous and non-monogamous relationship structures can help to improve mental health outcomes for clients engaging in varying relationship styles.

To best explore the lived experiences of jealousy across these relationship structures, the authors selected interpretative phenomenological analysis (IPA). IPA was developed largely within the health psychology field, and as such, it is well suited to an intimacy-sensitive study such as this. The method itself aims to make sense of people’s meaning-making processes and outlines rigorous steps to ensure the ethical recruitment of participants. The present study is an exploration of jealousy from the lived experience of both monogamous and consensually non-monogamous individuals. Though the present study does not draw from a clinical sample, the results may be of value to clinicians serving non-monogamous populations. The results of this study also help to address the gap in the literature exploring how the affective state of jealousy might impact mental health differently in various relationship structures.

## Method

Interpretative phenomenological analysis (IPA) was selected for its robustness in supporting in-depth investigations into idiographic psychological phenomena (Smith & Nizza, 2022). Study procedures, including participant sampling, data collection, and data analysis were consistent with the IPA method throughout. IPA prioritizes a relatively small and homogenous sample size, selected through purposive methods. This allows the data to illuminate a specific experience and minimize confounding factors (Smith & Nizza, 2022). Data collection, ethical concerns, and all study procedures were approved by the institutional review board of Westfield State University, approval number 21/22–047.

### Participants

Consistent with the parameters of IPA (Smith & Nizza, 2022), 11 cis-gender female participants were selected for the present study. Additionally, given the emphasis on homogeneity as outlined by IPA (Smith & Nizza, 2022; see also Smith et al., 2021), only cis-women over the age of 25 living in the United States were included in the present study. The initial call included data-gathering for all genders, though this study utilized only the women’s data. Individuals selected their gender identification as man, woman, or non-binary person during the initial recruitment survey. Transwomen were not included in the present study only because none responded to the call for participants.

The age of 25 was selected to allow the research team to focus more specifically on the post-adolescent experience of relationships. Cis-women living in the United States were chosen to mitigate the role of culture that might explain observed differences between participants. See Table 1 for a summary of participant demographic information. Participants were split into two subsamples, one which included self-identified monogamous participants (*n* = 6) and the other which included self-identified consensually non-monogamous (CNM) participants (*n* = 5). Participants did not have to be actively participating in multiple relationships at the time of the interview in order to qualify for study participation. Precedents for qualitative comparisons between groups in this way are documented in the empirical literature (e.g., Lysaker et al., 2015; Morrison et al., 2017). Inclusion criteria for the monogamous sample included: (a) identifying as monogamous; (b) having never experienced CNM (defined as the intentional mutually chosen decision to have more than one intimate partner simultaneously); (c) having experienced some substantial recognition of jealousy at some point in their romantic relationships; and (d) having an ability to communicate their experiences to the study interviewer. The ages of the monogamous sample ranged from 28–54 (*M* = 41.0, *SD* = 10.24). Inclusion criteria for the CNM sample included: (a) identifying as non-monogamous or polyamorous for at least 2 years; (b) having experienced jealousy at some point in their romantic relationships; and (c) having an ability to communicate their experiences to the study interviewer. The ages of the CNM sample ranged from 34–54 (*M* = 44.2, *SD* = 8.90). The ages of the overall sample ranged from 28–54 (*M* = 42.45, *SD* = 9.32).

**Table 1.** Participant demographic information (*N* = 11)

Participant Number	Relationship Structure	Age	Race	Orientation
M1 Madeline	Monogamous	28	Asian American	Heterosexual
M2 Mandy	Monogamous	54	White	Heterosexual
M3 Mae	Monogamous	45	Asian American	Heterosexual
M4 Misha	Monogamous	36	White	Bisexual
M5 Marcie	Monogamous	33	White	Bisexual
M6 Melissa	Monogamous	50	White	Bisexual
C1 Claire	Consensually non-monogamous	34	White	Het-flexible
C2 Cameron	Consensually non-monogamous	51	Black	Pansexual
C3 Cora	Consensually non- Monogamous	46	White	Bisexual
C4 Christen	Consensually non-monogamous	36	White	Het-Flexible
C5 Carolyn	Consensually non-monogamous	54	White	Bisexual

One participant in the CNM group (Carolyn) warrants special note and her situation raises a complicated issue in collecting data about relationship structure. Carolyn was unique among the CNM group in that she explicitly considered herself polyamorous, yet a partner of hers did not adhere to the central principle of informed consent with his spouse. The authors chose to prioritize Carolyn’s identity over the non-disclosure decisions of her partner. However, some interesting alignments appeared between Carolyn’s experiences and the monogamous participants, and notable differences appeared between Carolyn and the other CNM participants, which are described in the results section.

### Interviewer and Auditor

The first author’s positionality is as a queer, cis-gender White woman who is a polyamorous, married parent. She is certified as a sexuality educator and trained in analytical psychology. She had prior experience with IPA methods, specifically in the study of jealousy, and served as the interviewer and analyst for all interviews. Consistent with a constructivist approach, the interviewer emphasized the participants’ meaning-making process during the interviews, asking probing questions following the phenomena reported by the interviewees.

The second author, who was not involved with data collection or data analysis, was a monogamous, bisexual, cis-gender, White male psychologist with experience in multiple qualitative methods. The third author was a monogamous, heterosexual, cis-gender, White woman in a relationship with her partner of seven years. Her role as study auditor was consistent with IPA and deliberately integrated into the study to mitigate any biases that may have been present for the first author during data collection and data analysis. She was a clinical psychology graduate student who completed an audit of the analysis, coming to a consensus with the first author after all interviews were conducted. She had prior experience with qualitative methods, specifically with consensual qualitative research.

### Procedure

All study participants were recruited by general participant calls sent to an online listserv for sex therapists, educators, and counselors, and two publicly listed online (Facebook) communities devoted to discussing relationship issues. The first author used three different recruiting scripts: one for email or direct message for group moderators to use with participants, one for posting on large relationship forums on the internet, and one for email or direct message to send directly to potential participants. In order to collect participants from the appropriate demographics, the calls were sent out to groups where monogamous or non-monogamous people would typically be found. Participants were not offered compensation of any kind for participation.

The first author utilized a random number generator to select participants from the individuals who responded to the call via an electronic survey. The survey included items that helped to screen for suitability for the study, collected basic demographic information, and informed the participants of the length and timing of the research interview.

Participants were selected for 60-minute semi-structured interviews held via Zoom teleconference. Each interview was video-recorded and transcribed. Every participant provided informed consent regarding how their interview recording would be used and stored. Additionally, the participants were informed about the provisions for anonymity and confidentiality of the data collected.

The interview protocol was structured in alignment with the goals of IPA to have a rapport-building opening phase followed by questions designed to elicit highly detailed reports of participants’ feelings, thoughts, and actions when they experienced jealousy in the context of their romantic relationships. The semi-structured interview protocol can be found in Appendix A.

Rapport was established by the interviewer through the consistent use of active listening skills. The interviewer focused on the nuances of the participants’ verbal and non-verbal responses. In alignment with the goals of IPA, participants were encouraged to provide detailed responses to open-ended questions. The interviewer utilized probing follow-up prompts to encourage the participants to share their experiences as fully as possible. At the close of the interview, participants were informed of the purpose of the research study and their rights as consenting participants. Participants were reminded of the general support resources available to them in case they required post-interview support.

### Data analysis

The transcripts were analyzed by the first author using IPA, a qualitative method that involves a detailed exploration of participants’ personal accounts and experiences. This method of analysis allows simultaneous consideration of the shared and unique perceptions participants have when discussing a particular topic (Smith & Nizza, 2022).

Initially, the CNM sample data were collected, analyzed, then audited. The analysis was performed iteratively, in successive rounds of reading and review of the recorded video interviews. The analyzing researcher made exploratory notes, capturing her initial reactions to the texts. The exploratory note taking process was continued by making descriptive, linguistic, and conceptual notes about the content as she iteratively worked through the interview transcripts. Non-verbal communication cues such as facial expressions and hand gestures were included in the descriptive notes.

The next phase of analysis proceeded by deriving succinct experiential statements from the exploratory notes. Each experiential statement summarized both the content the participant shared and the personal psychological significance interpreted by the first author during analysis of a segment of the interview; this process is essential to the IPA method. Experiential statements are intended to capture both the explicit information shared and the richness of the participant’s narrative meaning in context of their entire interview.

Experiential statements were then clustered and examined to establish broader experiential themes (Smith & Nizza, 2022). The coder utilized a constructivist lens throughout the analysis, focusing on the ways the interview texts showed participants making meaning out of their lived experiences.

Next, the monogamous sample data were collected, analyzed, then audited, and that analysis was done in the same way as the CNM sample. All analyzed transcripts were then compared for within-group patterns and between-group differences.

To ensure the analysis was accurate, the third author served as the data auditor. The audit process entailed a comparison of the themes and subthemes applied to the interview excerpts. There was a brief discussion between the first and third authors in which the auditor agreed with the original analysis of the themes and subthemes. Additionally, all discrepancies of experiential statements between the analyst and auditor were discussed at length and addressed until both agreed on a consensus. These discrepancies were found to be rooted in the third author’s limited experience in how CNM participants utilized relationship terminology differently than monogamous participants did. All discrepancies were resolved through discussion and clarifying explorations of the vocabulary established during the literature review.

## Results

The purpose of this study was to describe the lived experiences of jealousy in monogamous and CNM women. All participant names presented in the results are pseudonyms. For ease of reading, all monogamous participants have been assigned names beginning with the letter “M” and all CNM participants have been given names beginning with “C”. Demographic information is located in Table 1.

The results demonstrated that jealousy is a complex emotional experience. The way participants made sense of jealousy was influenced by the relationship structure they participated in. Initial coding of the 11 manuscripts produced approximately 1,471 exploratory notes which yielded 91 subthemes. The final analytic process rendered four group experiential themes: jealousy’s somatic sensations, jealousy’s accompanying emotions, jealousy norms and beliefs, and strategies for managing jealousy.

**Figure 2.**
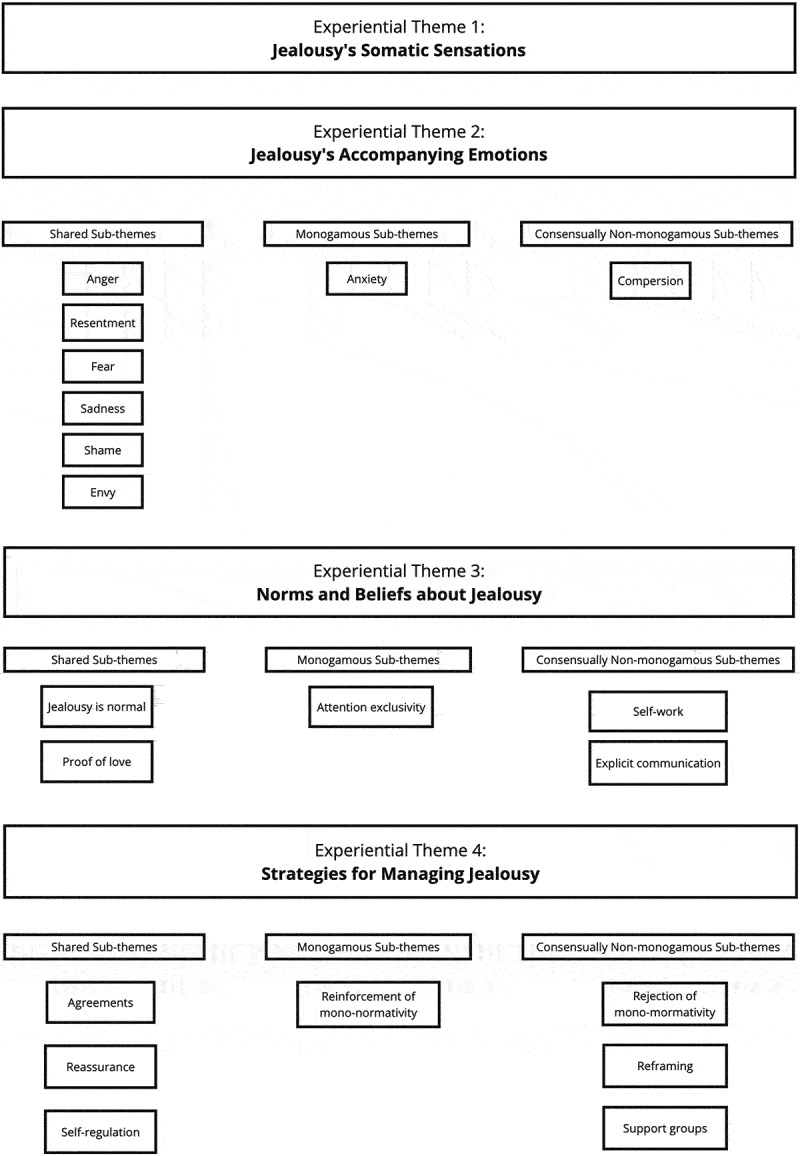
Experiential themes 1–4.

The subthemes pertinent to each group experiential theme were further organized into those shared by both monogamous and CNM relationship structures, those specific to monogamous relationship structures, and those specific to CNM relationship structures (see Figure 2).

To help elucidate the study findings, qualitative exemplars pertinent to final sub-themes are provided below to keep the interpretations grounded in the participants’ own words. Exemplar quotations included below have been lightly edited for clarity and brevity (e.g., removal of filler words and repeated sentences), while maintaining the integrity of the participants’ language. Exemplar quotes that were sufficiently rich could be grouped into more than one experiential theme.

### Experiential theme 1: Jealousy’s somatic sensations

Across both groups, participants described somatic sensations accompanying the emotional experience they identified as jealousy. All but one participant were able to identify one or more sensations, including several located in the belly: “stomach knots”, “nauseous”, “sick to my stomach”, “shooting pain in my gut”, and “a lurching feeling”. Participants also mentioned sensations in their chest: “heart beats faster”, “pain in my chest”, “heat in my chest”, and “it hurts in my chest”.

Madeline felt the intensity of her jealousy through multiple bodily sensations:

It’s definitely like being nauseated, but like my s-stomach was just turning, like almost a sort of seasick feeling. My, I think my head was kind of whirling, too. Like I just felt like, um… I don’t know. I was like, I don’t know, like a big stone in my stomach.

Christen described her jealousy as having a notable embodied quality, even as she worked through the mental and emotional components of a jealousy-inducing situation:

I think it’s the physical reaction. You know, you get like the … your heart beats faster, you get kind of a sinking feeling, or it’s definitely an actual like physiological reaction.

Claire noticed how the somatic sensations of constriction were mixed with other challenging physical and mental experiences:

I would say my body feels tight. Um, I have maybe racing thoughts. I, um, it can manifest as sleeping … poor sleep, lack of focus or concentration on other, like work- distraction. And then sometimes I’ll have this like sense of urgency.

The ability to identify sensations made it clear to participants that there really was something going on; whether that suspicion was warranted or not, it gave them the impetus to evaluate the current state of their relationship. Sensations were tied to the emotions reported by the participants, ranging from mild to overwhelming across participants.

### Experiential theme 2: Jealousy’s accompanying emotions

Jealousy is understood as either a simple emotion, irreducible and marked by its own specific motivational aims (Buss, 2013; Chung & Harris, 2018) or as a complex emotion, consisting of two or more other affective states (Mogilski et al., 2019; Pfeiffer & Wong, 1989; White & Mullen, 1989). Regardless of whether jealousy is conceptualized as simple or complex, the data revealed that jealousy is described by participants as accompanied by a host of other emotions when it arrives. Each individual noticed a unique array of emotions in different intensities during various times when they described themselves as jealous. The subthemes in this study represent the distinctive emotions most frequently named by participants: anger, resentment, fear, sadness, shame, envy, anxiety, and compersion.

### Anger

Participants reported a mix of emotions, with different expressions of anger being the most common among them. Every participant described some level of anger connected to their experiences of jealousy, though for the CNM participants, there was a sense that the anger dissipated as they learned to manage jealousy actively over time.

Madeline, a monogamous participant, recalled her anger as being bigger than any single incident of feeling wronged: “There was a lot of anger towards him that was probably also a projection of all of the guys that have screwed me over”. She also had strong feelings that jealousy in a “healthy amount” was a useful indicator that she and her partner were appropriately attached.

Mandy had a much more repressed tendency with her jealousy-related anger:

I realize I did not express any anger. It was probably … something within me, you know, like, what can I do? Why am I not enough? Wanting to believe that he was, was not going to do it [cheating behavior] again, and so it was like, okay, you know, just push the feelings down and continue. And there were some tears, but I never really expressed anger… until, you know, after the third episode (*laughs*). Even then, it, there was, it wasn’t until … it took me about a year to actually express anger about it. You know, we did a little couples therapy and then that didn’t seem to do much and I just continued on my own and, you know, finally realized I have not expressed any anger and I’ve got this resentment.

Mandy’s anger was apparent during most of the interview, and the emotional content brought tears at times, yet she was also aware that her lack of willingness to express her anger had potentially delayed finding a path through the jealousy toward more clearly negotiated relationship expectations.

Christen, a CNM participant, said she “would get angry and very insecure, but especially a lot of anger over it” especially in the early months of practicing non-monogamy. Similarly, Cora had very intense feelings of rage early in her CNM marriage, and she believed it was “definitely a fight or flight kind of response; usually, fight for me (*laughs*)”. Cora also noticed what she called jealousy most prominently when her husband’s attention was directed at his computer, which was a source of relational connection for him to other partners, potential partners, and friends. This brought out strong aggressive emotion: “There were times when I fantasized about throwing that computer on the ground, you know?”

### Resentment

One participant from each of the two groups described feeling resentment connected to jealousy. Each of these participants also noticed that the resentment seemed connected to unexpressed anger at their partner. Carolyn described feeling resentful of having to manage jealousy. Mandy was more effusive about the way jealousy-fueled anger festered into resentment:

After the third episode (*laughs*). Um, and, even then, it, there was, it wasn’t until … it took me about a year to actually express anger about it, um. You know, we were in, we did a little couples therapy and then that didn’t seem to do much and I just continued on my own and, you know, finally realized I have not expressed any anger and I’ve got this resentment. Some of it is resentment of, uh, you know, I’m resentful of myself, like why didn’t I say something, you know, why did I continue, uh, you know, making his lunch every day, you know, st- stupid things like that, where, you know, I’m like berating myself, like, “Oh, well, maybe if you would have done, expressed this, then, you know, it wouldn’t have happened again”, or, you know maybe we wouldn’t be together, so.

### Fear

Fear of loss or change to their relationship status was described by many participants across both the monogamous and CNM groups. For example, Melissa stated the following:

Stories I have in my head about what could happen that I’m freaking out about. He’s like, you’re not actually basing this off of things that have occurred, although other things have occurred that have led to this immense feeling of- of fear.

Several participants concurred with Melissa, noting that jealousy often had a fearful tone. The monogamous individuals tended to describe their fear in more concrete terms of being afraid they would lose their partner to another romantic interest. Several CNM participants noted that they had experienced fear during the early shift from a monogamous to CNM relationship structure but that it eased as they adjusted to their new relationship paradigm.

As described in the participant section, the CNM participant who described fear most frequently, Carolyn, was in a secret relationship with one of her partners. She described intense fear when describing her most important intimate relationship. The practices of transparency and disclosure typically centered in CNM relationships, which was not part of Carolyn’s relational experience, had an impact on the way she experienced jealousy. While a typical CNM relationship is based on the full knowledge of all involved parties, Carolyn identified as polyamorous *and* had agreed to be the secret partner to a married man. She described ongoing intense jealousy as well as deep desire to end the secrecy and be included in her lover’s whole life. Carolyn processed her painful experiences of jealousy through talk with a sibling who knew the circumstances of the relationship, an extensive journaling practice, and expressive art in the form of poetry.

Fear sparked a sense of urgency for several participants, evidenced by reports of distraction and pressure to find a resolution to their emotional state as quickly as possible. Claire described: “[Jealousy] presses for attention- right? So, immediacy is a good word. And like the urgency of it can be significant”. On the other hand, Christen intentionally chose not to engage the sense of urgency when it arose:

It’s easy to compartmentalize or just put aside and say, “I will think about this later. I know that it’s going to be too distracting now whether it’s because I’m at work or whether it’s because I need to be present”. So it’s easier to say, “It doesn’t need to be fixed right away. There’s no immediacy to this, it can wait. Put it aside and let it be”.

Each participant described ways of managing their fear response which will be discussed further in the jealousy strategies section.

### Sadness

Sadness was mentioned by three monogamous participants and one CNM participant. Interestingly, sadness was not described with particular depth by any of the participants. When describing their jealousy each simply listed sadness but then pivoted into richer descriptions of other emotions, such as anger, shame, and resentment. Carolyn offered a piece of poetry written in response to her jealousy that evoked images of sadness tinged by anger and loneliness.

### Shame

Participants from both groups discussed how shame made it more difficult to work with their jealousy. Carolyn paused and considered how shame distanced her from her beloved:

I won’t explicitly share my jealous feelings with him because I’m embarrassed by them. Sharing feelings of jealousy with someone, especially when those jealousy feelings involve the person you love, is a bit like exposing one’s tender underbelly. For me, to have feelings of jealousy are shameful: I’m ashamed to have them.

Melissa described having researched jealousy to try to understand it better specifically because of the intense shame it evoked in her. She believed that there was “cultural shame around it [jealousy] just embedded” into all of us. Melissa found that working with jealousy was made much more difficult because “there’s a lot of shame around it. You know, everyone says how horrible jealousy is”. She did not feel comfortable naming that she was jealous to her friends or boyfriend until she researched it and decided that it was a normal, if challenging, emotion.

### Envy

Envy was noted by several participants in each group. A word often conflated with jealousy, envy is the emotional experience of longing for someone else’s advantages. In common usage, many people freely interchange the words jealousy and envy, but one compelling reason to differentiate between them is that they can appear simultaneously and when they do, the differences are important factors in working productively with each emotion. Carolyn clarified her understanding of the words:

For me, the two are not synonymous. I think it’s a difference between power and empowerment. I think “jealousy” is more about feeling that someone else has something you want and you have no control to change it, and “envy” is wanting what someone else has, but having the power to change it.

This aligned with Carolyn’s descriptions of jealousy, which centered around a profound sense of helplessness to affect the outcome of her love story.

Christen employed knowledge of the difference between jealousy and envy to more effectively meet her relational needs:

If I’m feeling neglected or I’m feeling like I need more time, or I want to be able to go out and do something special, I will bring that up and say, you know, “I’m not angry at this, but I realized I’m not getting that time [that I want] so I want to do this”. So yeah, I will bring it up, if it’s something like that. If they’re on a date to a movie I want to see or at restaurant I want to go to, then it’s just like, “Okay, that’s just a little envious and I might not bother bringing it up”.

Christen went on to share how she chose to handle envy by going out with friends, making time to do things on her own, and by remembering that she did not need to have the same experiences with her husband that his girlfriend had because she wanted different things. She was quite explicit in this description, noting that his girlfriend had different sexual interests than she did and that she appreciated getting to a place of feeling genuinely happy that his needs for certain acts were met by someone who truly enjoyed them rather than feeling obligated to participate in sexual play she did not find particularly interesting.

### Anxiety

While fear is an emotion focused on known external danger, anxiety is an emotional response to an unspecific threat (Steimer, 2002). Fear was identified by participants in both groups, while anxiety was reported only—and universally—by the monogamous participants. Anxiousness was described when participants were asked about physical sensations, the timing and context of their jealousy, as well as when they were asked to define jealousy in their own words. Anxiety was almost synonymous with jealousy across the monogamous interviews.

### Compersion

Compersion, the range of positive emotions that may be felt in response to seeing another person experiencing joy, is not yet a common word used in monogamous settings. Though compersion can be understood as an antonym of jealousy, it is also common to feel compersive and jealous simultaneously (Thouin-Savard & Flicker, 2023).

Among the monogamous participants in this study, only two knew the existence of the word compersion, whereas the entire sample of CNM participants knew of the word. Though neither of these monogamous participants reported experiencing compersion themselves, both were employed as therapists and worked with couples. When asked if they knew of a word that meant the opposite of jealousy, these two monogamous participants each recalled learning the word from CNM clients. None of the monogamous participants, nor Carolyn, the CNM participant with a non-typical CNM relationship, described experiencing compersion when elaborating on their jealousy. It may be challenging to articulate and intentionally nurture compersion if one does not have an awareness of its existence or potential.

Four of the five CNM participants mentioned having personal affective experience of compersion during their descriptions of jealousy, and three noted that compersion took some conscious cultivation on their part. Cora said, “It’s been a learned skill. It’s, it’s been, over the years as I’ve become more secure, I’ve been more able to experience compersion”. This aligns with research by Thouin-Savard (2021) which described compersion as both attitudinal, that is, fostered through intentional thoughts and actions, and embodied, which may be experienced as spontaneous emotional affect.

Several CNM participants described compersion, ranging from a relatively mild experience of warmth to an active excitement for their partner’s upcoming dates and sexual play with others, particularly when they felt connected to their partner, secure in their agreements, and in good spirits with their partner’s other partners (also called metamours).

### Experiential theme 3: norms and beliefs about jealousy

The context in which jealousy occurs is relational (Kellett & Stockton, 2021). Depending on the type of relationship structure the participants practiced, they held different norms and beliefs concerning jealousy. These norms and beliefs set the stage for the meaning participants made of their jealous experiences.

### Jealousy understood as normal

There was universal agreement among participants that jealousy is a normal part of the human experience, albeit a challenging one. This willingness to accept jealousy as a normal affective state empowered Melissa to self-advocate for her relational needs:

Even my boyfriend said in the very beginning of some of this coming up, you know, j- jealousy is not attractive or whatever. And, um, and then I pointed out to some stuff, saying this is a perfectly relevant and normal emotion for people to have. It doesn’t … Don’t shame me for this, you know. You’ve told me, you know, you think she’s hot, so, of course, I might have some jealousy (*laughs*) around this person. So, um, yeah. So he’s- he’s backed off shaming me for it, which is good.

Normalization of the emotion served to empower Melissa to work on her jealousy consciously, though she struggled to get her partner’s full engagement in the process. Differences between partners in this way showed up in several interviews, with those who experienced some level of jealousy-shaming struggling more with it.

### Proof of love

Beyond just being normal, participants in both groups considered that jealousy functioned as a source of information about their relationship, though the meaning of that information differed between the participants, not associated with their relationship structure.

A monogamous participant, Madeline, described how jealousy supplied crucial information that her relationship was appropriately secure. When asked what role she believed jealousy should play in a close relationship, Madeline clarified, “I would be more concerned if I wasn’t jealous, like I wasn’t attached enough”. However, she also went on to describe several instances where she struggled to feel “psychologically secure” due to her heightened sensitivity to threat from anyone who she perceived as interested in her boyfriend, whether romantically or as a friend.

Misha had a different angle on how jealousy could serve as proof that her monogamous relationship was secure enough to even enjoy jealousy, at least in small amounts and in non-threatening situations:

I think it can be, like, fun if … the dose, like dose, makes the poison. So like a little bit of jealousy. Like I was wearing a, oh, a swimsuit. We were at the pool with the kids, like not, not a very sexy situation. But it was showing my cleavage, and I saw, like, an old dude staring at it. And that did nothing for me, but the thing that I liked about that was then telling my husband about it later, and having him be like a little bit jealous. So I think it can be fun and sexy in a like, getting in touch, especially in like a long term relationship where’s there’s not a lot of tension, like, tension anymore. Or there’s not a lot of, um … things are very safe and secure. So adding a little bit of risk, a little bit of danger, can be, like, fun.

One CNM participant, Claire, conceptualized jealousy as valuable information as well:

Like, we should listen to what our emotions are telling us. So what, what is jealousy telling me, right? So, jealousy is telling me that I care a lot about … I care a lot about this person. I care about them so much that I’m worried about losing them. I care about them so much that I want them to think I’m really cool. Uh, you know, I care about them so much that I want a lot of their time.

Claire also explained that it was her responsibility to “parse out” and to navigate her next steps so she did not act on that jealousy information with a sense of entitlement to someone’s attention, body, or time.

### Attention exclusivity

When asked to describe jealousy, all of the monogamous individuals indicated that they had an expectation that their partner would spend the majority of their relational attention on only them. Each also noted that they did encourage friendships, but their comments suggested that there is a certain quality of attention that is appropriately reserved for only one’s monogamous partner.

Mandy experienced the lack of attention as directly related to her experience of jealousy:

I guess it’s that, that feeling of, um, somebody’s taking my spot… somebody is getting my attention. He’s very charismatic and flirtatious, which drew me to him in the first place, um, and he, you know, is like that with everybody. When we initially started dating, uh, uh, you know, it was directed towards me.

Madeline similarly recognized jealousy when she “didn’t feel like I was prioritized”. She also noticed that retroactive jealousy, that is, jealousy about her partner’s past lovers, continued to cause significant amounts of jealousy in the present even though they could not impact the actual physical attention she received now: “I was jealous of the fact that like, those women got his physical attention, you know what I mean?”

Melissa expressed that her jealousy was most difficult to manage when another woman in their friend group was paying attention to her partner, particularly since she had earlier experiences that led her to believe that a loss of his attention was dangerous to her committed relationship.

This is juxtaposed against the CNM participants who described a different expectation around the exclusivity of attention. Claire interpreted attention as something that did not need to be held exclusively, stating, “Jealousy can be, um, this idea of ownership. Or like entitlement to someone else’s, um, physical, uh, attention or emotional attention, or time, um, or body”. This sentiment was mirrored by all of the other CNM participants, though each also acknowledged that it took some time and effort to shift their perspective around how attention works in close relationships.

### Self-Work

Though jealousy exists within a social context, and thus could be easily understood to be a problem that must be solved by those one is in a relationship with, CNM participants universally acknowledged that responsibility for recognizing and managing jealousy belongs first to the individual experiencing jealousy. Cameron put it simply: “Usually, jealousy is a me thing”.

Cora commented on her eventual acceptance that jealousy, and other big feelings, required her to do both the inner recognition work and the communication work:

I grew up in an environment where showing your emotions and feeling your emotions was not encouraged at all. It was actually dangerous. And so I had to learn as an adult to identify my emotions and express them in a way that was appropriate.

She noted that this process had taken between ten and twenty years of effortful self-work.

Carolyn struggled to feel seen as a whole person because her partner prioritized his other partner with the explanation that he was committed to protecting his spouse’s “pure heart” even from his own deceptive relationship practices. As Carolyn’s jealousy reached overwhelming levels two years into their relationship, she began to do some deep inner work on accepting her negative or shadow qualities. Her tools of choice were Buddhist philosophy and acceptance. She described how this had positively impacted her life:

I think human beings, all human beings, have a dark side. And I think that’s okay. I think it’s actually a good thing for our creative selves and we need to acknowledge our dark side, accept it as part of who we are; not push it away and pretend that it doesn’t exist.

### Explicit communication

While monogamous participants of course engaged in communication with their partners, there was a more pronounced emphasis on explicit communication about jealousy among the CNM group. Four of the five CNM participants stressed explicit communication as a baseline norm that required specific effort.

Each of these five had intentionally engaged in explicit communication about jealousy. They conversed about jealousy’s meaning and purpose directly with their partners. Claire made it a regular practice, stating, “I’ve had multiple processing conversations about jealousy with multiple partners including my spouse, because it’s one of the things that comes up … that we discuss openly”.

Cameron, who prioritized her inner work with jealousy shared one of her core self-awareness checks so that she could get clear about her needs before bringing the issue to her partners:

It helps me to say, “Okay, what am I jealous of?” Because there’s a lot of things you could be jealous of, right? You can be jealous of somebody’s emotional connection, you can be jealous of time spent with someone, or more things, So for me, identifying particularly what I’m jealous of helps a lot to-to process it. Yeah. I wanna be specific.

Cora shared how she learned to notice the signs that jealousy was rising, noting, “Like I suddenly start feeling mad and I can’t figure out why I’m so mad all of a sudden”. Over time she described learning to turn to communication tools more quickly:

I go straight to the other person now. I’ve learned that I build it up into my head to be much worse than it is almost all the time. And so it’s better to just go talk to the other person before I stress out. They are very good at listening and they’re both very good about, you know, hearing me and accommodating whatever it is that I need. You know they’re both very reassuring and very open to communication.

All but one of the CNM participants also normalized talking about jealousy in their friend circles and support communities. The one who did not report talking about jealousy with others very much, shared that they had struggled to find community spaces, but that they wanted to talk about jealousy and relationship struggles more among their friend groups.

### Experiential theme 4: strategies for managing jealousy

The norms and beliefs of each group revealed a set of strategies the participants used to manage their personal experiences of jealousy. The norms and beliefs of the two groups appear to have influenced the development of different strategies or differing ways of utilizing said strategies.

### Agreements

Any consensual relationship could be understood as an agreement between two or more people to be connected. Beyond that most basic definition, agreements serve to create the expectations of a relationship. Both groups spoke of their relationship agreements, though there was a considerable between-group difference in how agreements were formed and the level of explicit communication utilized to maintain the agreements.

Mae, in concurrence with all of the monogamous participants, did not have any formal relationship agreements. Asked if she had any rules or agreements that helped her manage or avoid jealousy she said, “Um, no, I don’t think so. Nothing that has been verbalized”. She also considered the default monogamous expectations to form the basic parameters of her relationship agreements. When asked what her monogamy entails, she responded:

We’ve never talked about it? I think it was when we first got back together, I think it was just like an expectation that we wouldn’t see or be with anybody outside the relationship, whether it be sexual or emotional, um, make, I did voice to him that you could be cheating on me even if you’re not having sex with somebody. If you have an emotional relationship, I consider that cheating.

Marcie also outlined a reliance on monogamous norms versus explicitly negotiated relationship agreements:

Oh, the, the agreement of monogamy. Oh, yeah. We, um, yeah, that we would be just, you know, all of the things just with each other… um, emotional, sexual, all of those things … Um, but it’s those types of things that we agree, like, this is the primary person where you get most of the things from.

Claire used explicit communication in negotiating her agreements right from the start. When asked about agreements she stated, “I was pretty intentional about the way I wanted to structure my life. And literally on our first date I said it [what I wanted]”.

Other CNM participants echoed Claire. They universally described making agreements in order to explore non-monogamy consensually. These agreements varied in style and content, but all the CNM participants mentioned ways they had negotiated and renegotiated with partners over time in order to handle the jealousy that came up through their expansive emotional, sensual, and sexual connections.

### Reassurance

Reassurance that the relationship is strong was a common thread across groups, in both positive and negative forms. Madeline described how the absence of reassurance caused significant distress leading to a breakup, stating, “I have a lot of close, platonic friends too. The difference here is that, um, like he wasn’t able to reassure me, you know, that we were okay”.

Mandy discussed the benefits of receiving proactive reassurance for her feelings of insecurity:

You know, that’s that, that inside fear, but then, when I think about it rationally and, you know, what he says is, you know, “I’m not looking for another relationship-” … you know, so, um- Um, he does offer [reassurance], uh, before I ask, yeah, and if I, you know, if I’m … I, I’ve been feeling insecure.

She struggled because she felt needy if she wanted reassurance, which she described as uncomfortable and confusing.

Cora made progress with her jealousy by noticing inexplicable emotional reactions early so she could take immediate action asking for clarity and reassurance:

I suddenly start feeling mad and I can’t figure out why I’m so mad all of a sudden. I go straight to the other person now. Um, I’ve learned that I build it up into my head to be much worse than it is almost all the time. And so it’s better to just go talk to the other person before I stress out. Both my husband and boyfriend are very good at listening and they’re both very good about hearing me and accommodating whatever it is that I need. You know they’re both very reassuring and very open to communication.

### Self-Regulation

Self-regulating activities were utilized by all participants, in a variety of forms. Mandy, like several others, relied on somatic tools:

Part of it is like grounding, so I’ve been doing some yoga, so just trying to kind of breathe and, and meditate in a way, I’ve been doing that as well. Um, but the other things like I, uh, I realize that I’m kind of doing like childish things, things that I never did as a kid, like learning how to do a handstand.

Other participants, like Cameron, approached their self-regulation through self-inquiry:

It helps me to say, “Okay, what am I jealous of?” Because there’s a lot of things you could be jealous of, right? You can be jealous of somebody’s emotional connection, you can be jealous of time spent with someone, or more things, but it really helps to be clear on what I’m jealous of so I can ask for what I need around that specific thing.

However, several participants found that rumination led to more difficulty managing their jealous reactions unless they intentionally engaged self-regulation practices. In other words, jealousy was experienced as significantly dysregulating enough as to inspire people to employ specific self-regulation interventions. This was especially notable in the participants who did not feel comfortable talking about their jealousy directly with their partners.

### Reinforcement of mono-normativity

Norms of each group also served as the basis for jealousy management strategies. Many of the monogamous participants expressed that their jealousy was handled well by choosing monogamy with someone they feel is trustworthy. Therefore, when jealousy showed up, rather than searching for a solution within themselves, the monogamous participants largely expected the norms of monogamy itself to curtail the activities that inspired their jealous moments.

For Misha, the mono-normative expectation was that she would be centered in her partner’s life, and so long as she had that, she experienced little jealousy:

I don’t think I’ve really thought about it quite like this before, now that I’m saying it, but I think I feel jealous if I feel like, not preferred or not chosen. And if I feel chosen, and also, like, in the loop and in the know, then I don’t care. But if I feel like there’s something going on without me, or I’m left out of it, or, yeah, then that is when I start to feel insecure and more jealous.

However, the usefulness of mono-normative standards was not unanimous. Mandy struggled with a partner who would not adhere to the mono-normative expectation of not “sexting” (sharing sexually-toned, flirtatious conversations via text or messaging app) with other women. This caused significant and persistent distress for years. Mandy seemed caught in a conundrum because her ostensibly monogamous relationship did not provide a sense of secure chosen-ness, yet they also did not establish any of the normative behaviors for non-monogamous partnering.

### Rejection of mono-normativity

Members of the CNM group consistently expressed that they relied on their rejection of mono-normativity to bolster their jealousy management. Christen, Claire, and Cameron each described how they found themselves at odds with mono-norms early on in life.

Christen realized that her inclinations toward multiple partners had always left her feeling ill-served by the rules of monogamous coupling:

I don’t think I’ve ever had a relationship, a boyfriend, no matter how serious it was, that I haven’t cheated on. I haven’t been able to place that boundary on myself and actually stick to it. I can’t just meet somebody and pull back whatever connection there might be- I want to form an actual deeper connection with people I meet. I’ve been realizing it was endemic to me because after I got married and had a great relationship and a great sex life, there I am … still making connections with other people. So, coming to terms with it. This is just who I am, this is how I relate to people. So I make relationships where I can be myself.

Christen did not want to cheat, nor did she wish to deny her capacity to feel multiple simultaneous connections just because she had chosen to marry. Learning the terminology and best practices of CNM gave her a way to be honest, negotiate with her spouse, and find a way to refrain from cheating. The desire not to cheat actually drove Christen’s explorations in CNM, and to reject the norms of monogamy in favor of consensually negotiated non-monogamy.

Claire leveraged her endemic desire to create relationship structures that work best for her, noting, “I’ve kind of always enjoyed the idea of my partner being sexual with other people”. Over time, this formed the core of her relationship values, which she used to navigate jealousy when it inevitably came along:

I value consensual non-monogamy. And so, because it’s really important to me I reaffirm, the values I have. Like, I’ll say, I don’t own my spouse. I’m not entitled to him, or his body, and I want to celebrate his sexuality. I want him to be able to embrace his sexuality in whatever way feels good to him so long as it’s safe and consensual.

Cameron never considered herself monogamous. One of her first relationships produced spontaneous compersion and a desire to share rather than hoard her partner’s sexual attention:

I was deeply in love with, and I just thought he was so beautiful, and so healing, and so … Just being with him was just so good for me. I wanted other people to experience it. And I just felt like everybody should have you. (*laughs*) Everybody should have you. And I don’t know where that came from. I don’t know where that came from. I don’t have any idea where that came from.

Cameron found she did not need to know where her compersion came from, she simply enjoyed it. She had made two significant attempts over the years to participate in monogamy, but when she adhered to monogamous norms rather than her authentic self-expression of love, her physical health suffered.

### Reframing

Several of the CNM participants had done extensive contemplation on the nature of jealousy. They had found it productive to reframe their jealousy as a useful indicator to attend to their relational emotions. Claire made meaning of her jealousy without granting it too much importance versus the host of other emotions she might experience in a relationship:

I think all humans feel something that could be called jealousy, whether it’s insecurity, whether it’s possessiveness, whether it’s fear of loss, um, whatever it is. Your feelings aren’t wrong, right? There’s nothing wrong with feeling your feeling. Um, it’s what you do with it. And the way that I reframe jealousy is, jealousy is a strong emotion, right? It can be. Like anger, or right. Right. And our emotions are telling us something, and we should … we should listen to that, in my opinion, right? Like, we should listen to what our emotions are telling us. So what, what is jealousy telling me, right? So, jealousy is telling me that I care a lot about … I care a lot about this person. I care about them so much that I’m worried about losing them. I care about them so much that I want them to think I’m really cool. Uh, you know, I care about them so much that I want a lot of their time. That’s all it’s telling me. It’s telling me that I care a lot about this person.

Reframing did not come easily in most cases though; each CNM participant also commented on the intentional time and effort it took to name and process jealousy and ease their experience of it.

### Receiving support from CNM peer groups

Several participants struggled with shame concerning their jealousy. Being able to talk about jealousy openly destigmatized the intense emotion that was frequently described as shameful.

Cora lived in a remote area without much local CNM community, so she turned to online support groups:

I had access to a lot of online groups where I could discuss polyamory over the years and discuss other people’s relationship issues with them and get an idea of how these things tend to work. That has been just invaluable, especially when there was a hard situation and I was having some emotions. It’s, it’s been invaluable having people online that I can just go to and talk to about it.

Even when there was no specific concern going on, Christen found that having a community, in her case local, in-person circles, helped her manage the complexity of jealousy along with managing a complex relational life: “It’s a small community that I’m a part of. I think it’s [jealousy’s] an open topic, definitely”. Claire also built an intentional CNM community, noting that she initiated and did some facilitating for the groups she was a part of, and though they were not huge, they provided a sense of safety while building less conventional relationship structures into her life. Cameron had multiple overlapping community resources. Once again, Carolyn was the outlier, having less access to community resources since she was in a relationship that was secretive. This impacted her sense of security significantly.

## Discussion

This study set out to explore the lived experience of jealousy among women who characterized their relationship style as either monogamous or CNM. Analysis of the findings revealed four experiential themes: “jealousy’s somatic sensations”, “jealousy’s accompanying emotions”, “norms and beliefs about jealousy”, and “strategies for managing jealousy”, each of which was organized into several subthemes. The subthemes were further classified into three types: subthemes present across the two relationship styles studied, subthemes found only in monogamous relationship styles, and subthemes found only in CNM relationship styles.

### Experiential theme 1: jealousy’s somatic sensations

Somatic sensations during jealousy were reported by all participants, suggesting that it is normal and perhaps even advantageous for jealousy to register as an embodied experience regardless of relationship structure or the strategies employed by individuals to enhance their sense of security.

### Experiential theme 2: jealousy’s accompanying emotions

#### Anger

Anger was reported by all participants, to varying degrees. This suggests that the underlying cause of anger during experiences of jealousy may not be related to the relationship style of the participants.

#### Resentment

Resentment was less frequently reported and was linked to anger in all instances; those who did feel resentful also emoted with tears and nonverbal indications of frustration while sharing their jealousy stories.

#### Fear

As stated earlier, jealousy is an emotional experience related to threat-detection in valued relationships (DeSteno et al., 2006). As expected, fear was noted by all participants across relationship structures when jealousy was present. This supports the notion that fear of change or interruption to one’s relationship is a critical way people identify jealousy. This is in alignment with established definitions of jealousy being a rooted in a sense of threat (White & Mullen, 1989).

#### Sadness

Sadness was also mentioned minimally, but across both groups. From their rich descriptive stories, it seemed that fear and anger overshadowed sadness for most of the participants.

#### Shame

Shame kept more than one participant from sharing their jealousy with others, thus making it more difficult to resource themselves to manage jealousy.

#### Envy

Envy, present in both groups, was also less frequently mentioned, which may have been due to the contemporary habit of using jealousy and envy interchangeably, despite their differences in the psychological literature. Those who did describe a clear differentiation of envy and jealousy had utilized that knowledge to work more advantageously with these murky emotions. Jealousy and envy can coexist. The image of the jealousy triangle (see Figure 1) may clarify why these two emotions may frequently appear together. The jealousy triangle consists of the jealous one, the valued other, and the perceived interrupter. When a perceived interrupter is understood to have valuable qualities that could draw the attention of the valued other, then the jealous one may also find themselves feeling envious of those qualities. This could potentially compound the intensity of the jealousy. On the other hand, envy was described as motivating to two participants, alerting them to make proactive changes to their lives. Experienced in a non-pathological dosage, envy was described as useful by these participants.

#### Anxiety

Surprisingly, anxiety was felt by every monogamous participant and none of the CNM participants. Anxiety, an emotion similar but not identical to fear, is understood to be future-focused and lacking specificity of threat source (Kazdin, 2000). It is possible that the act of intentionally agreeing to the presence or possibility of one’s partner having other people in their lives mitigates the experience of anxiety for CNM individuals because they have established clear paths to discuss the presence of so-called rivals to their relationship. It may be that they have a clear understanding of who could interrupt their relationship, whereas monogamous individuals may experience more subtle signals that their relationship is not as secure as they would prefer, leading to anxiety rather than a specific-target fear response.

#### Compersion

Compersion was reported as an active experience only by the CNM participants, versus the monogamous participants, two of whom knew of the word but had not experienced it personally. This could be attributed to the fact that the word is still making its way into the language and that it was coined for use within the CNM community, which means that it may still lie largely outside the monogamous lexicon and/or imagination.

### Experiential theme 3: norms and beliefs about jealousy

#### Jealousy as normal and proof of love

All participants expressed that experiencing jealousy was a normal part of relationships. Though all participants utilized jealousy as information about the state and value of their relationships, what participants believed jealousy meant about their relationship quality or stability varied. This suggests that it would be a valuable point of inquiry to encourage partners to describe what they believe jealousy’s meaning, value, or purpose is before further exploring the topic. Individuals may vary in their pre-cognitive commitments to jealousy’s meaning, which may impact the stories they construct about jealousy-related incidents.

#### Attention exclusivity

A belief specific to the monogamous participants was that there is a qualitative type of attention that is reserved for, expected from, and only experienced within the monogamous dyad. A breach of this expected attention-exclusivity was understood to be a violation of the core value and purpose of the relationship. This belief was not shared by the non-monogamous participants.

#### Self work & explicit communication

CNM participants reported specific commitments to ongoing, intentional jealousy processing both on their own and with their partners. Several mentioned seeing jealousy as an opportunity for self-work, albeit one they did not relish, that eventually resulted in an increased sense of capability in their emotional self-regulation. This was different from the monogamous participant’s reports which indicated jealousy was a topic of inner work and explicit communication only when jealousy erupted into their conscious awareness through a breach of exclusivity expectations. This seems to indicate an opportunity to strengthen relational resiliency across relationship structures through the normalization of jealousy discussions, self-reflection, and communication.

### Experiential theme 4: strategies for managing jealousy

#### Agreements, reassurance, and self-regulation

The jealousy management strategies observed across participant groups were agreements, reassurance, and self-regulation. Although these strategies were shared, there were considerable between-group differences in each of them. The CNM participants were more intentional and explicit in their agreements, while the monogamous participants tended to rely on unspoken or culturally-normative implicit agreements. The use of reassurance as a strategy to minimize jealousy-related discomfort also varied between groups. The monogamous participants tended to report reliance on their partner to self-monitor for behaviors that might inspire jealousy, while CNM participants reported asking for reassurance directly from their partner when they experienced challenging feelings like jealousy, which provided opportunities to apply relational tools as the feelings arise. Self-regulation emerged in a variety of forms, including breath work, meditation, and movement, and was a common strategy across participants for coping with jealousy, which is in line with the observation that the feelings related to jealousy are often experienced as somatically disruptive.

#### Reinforcement of mono-normativity

Reinforcement of mono-normativity was a strategy specific to the monogamous group. Mono-normativity in this context refers to the cultural standards and assumptions that define a romantic relationship as necessarily between exactly two people (Clardy, 2023). These social tenets are manifold, and participants in this group relied on them to uphold relational boundaries and in part to avoid potential experiences of jealousy. The definition and lived experience of monogamy can vary widely between individuals, including between monogamous partners (e.g., people may not have clear, explicit understanding of what monogamy looks like in practice and where the exact lines are that separate friendly behavior from cheating behavior). Reinforcement of mono-normativity was employed by the monogamous participants to prevent the experience of jealousy with inconsistent results. This strategy was counterproductive for some participants who did not feel comfortable bringing jealousy up directly with their partners. When reliance on mono-normativity has not prevented the experience of jealousy, and the monogamous tenets are left implicit, the differing understanding of the rules and guiding principles of the relationship can lead to misunderstandings and exacerbate the experience of jealousy.

#### Rejection of mono-normativity

The CNM group participants consistently rejected mono-normative expectations. This rejection took the form of identifying and modifying the features of a relationship that were rooted in monogamy and romantic/sexual/emotional exclusivity. It also included the practice of designing relationship structures that were intentionally different from monogamy; each CNM participant noted that this took time and effort. Having reconfigured their lives to de-center monogamous assumptions and expectations, they were able to more easily identify experiences of jealousy that they understood to be rooted in the tenets of monogamy. Once identified, the participants could reassess their perception of relational threat that underlies jealousy and alter their responses to the experience of that threat.

#### Reframing and support groups

The strategy of reframing jealousy was specific to the CNM group. Applying a lens of multiplicity to their experiences of jealousy allowed participants to look past the immediate threat response to the other information and meaning available from the experience. This allowed them to experience jealousy as a complex phenomenon with both positive and negative qualities. CNM participants were also able to access peer support from local CNM meetups, online forums, and organically generated CNM friend groups. This strategy gave them access to a context that allowed them to openly discuss topics, like jealousy, that might be stigmatized in other settings, and to share experiences and strategies for managing jealousy.

### Implications for practice

As outlined above, both similarities and differences between groups in experiences of jealousy emerged in the present study. This suggests that it is possible for conversations about jealousy to be included in the therapeutic environment as a constructive part of the process for both monogamous and CNM relationship structures. Understanding the way that relationship structure influences norms and beliefs would enable the clinician to more effectively discuss jealousy with clients.

Given this study’s findings, coupled with the extant clinical literature, the authors encourage clinicians to consider several strategies when working with jealousy across these populations.

#### Initiating jealousy conversations

First, clinicians might consider the role of jealousy in monogamous clients. For example, as noted above under the norms and beliefs about jealousy theme, all of the monogamous individuals in the present study indicated that they had an expectation that their partner would spend the majority of their relational attention on only them. Relatedly, under the strategies for managing jealousy theme, none of those participants addressed formal relationship agreements with their partners. These findings suggest potential “blind spots” in this sample and could point to assumptions or expectations that may need to be addressed in the therapeutic space. This is not to say that the experiences of these individuals necessarily need to be changed, but rather that clinicians might consider addressing these issues more explicitly when targeting relationship issues in therapy.

#### Relationship diversity awareness

Given the historically neglected positionality of CNM people, the authors would like to address potential strategies in this population more directly. Perhaps one of the most straightforward and immediate ways clinicians can improve their experiences and outcomes with CNM people is by familiarizing themselves with professional guidelines regarding the care for these individuals. For example, Division 44 of the American Psychological Association (APA;, n.d.) has established a Committee on Consensual Non-Monogamy, and its website offers a variety of resources for those interested in learning more about the CNM paradigm including publications, fact sheets, and points of contact. Relatedly, the APA Guidelines for Psychological Practice with Sexual Minority Persons (2021) also addresses best practices with CNM people. For example, Guideline 9 states, “Psychologists strive to be knowledgeable about and respect diverse relationships among sexual minority persons” (APA, 2021, p. 21). Both the rationale and application of this Guideline address issues related to CNM in this population, including steps to rectify the ongoing invalidation and worse therapeutic outcomes experienced by CNM people in mental health settings. Clinicians are encouraged to consider consultation with other professional organizations as empirical research and best practices in this population continue to evolve.

In addition to preparing “behind the scenes”, clinicians can actively change their approach when working directly with CNM clients. For example, the authors encourage clinicians to consider the role of jealousy through a multicultural lens. Specifically, a multicultural orientation (MCO) may be a particularly helpful way to conceptualize interventions with CNM clients. MCO can reflect a “way of being” with clients guided by clinicians’ values about the salience of client cultural factors (Owen et al., 2011). MCO consists of at least three components, including cultural humility, cultural opportunities and missed opportunities, and cultural comfort (Owen, 2013).

Each of these components can be considered when working with CNM clients. Cultural humility involves an other-oriented perspective on the part of the clinician characterized by respect, lack of superiority, and attunement regarding their own cultural beliefs and values (Hook et al., 2017). This stance of cultural humility when working with CNM clients may be particularly important given the historical and ongoing social stigma associated with this relationship paradigm. Cultural opportunities represent situations in the therapeutic environment in which a clinician might better understand a client’s cultural heritage (Owen, 2013). For example, when a CNM client discusses frustration with their partners, the clinician may explore the client’s experience of jealousy from a position of genuine curiosity, rather than assuming the client needs to change their behavior to conform to a mono-normative worldview. An invitation of this kind from the clinician allows a cultural opportunity to shift the content of the therapeutic situation to a topic that may not otherwise have been considered by the client (perhaps because they had not thought to address it, they imagined it taboo to discuss with their clinician, or numerous other reasons). An inability or unwillingness on the part of the clinician to invite the client’s consideration of a CNM perspective would represent a missed cultural opportunity. Lastly, MCO involves cultural comfort, which reflects the ability of the clinician to initiate cultural conversations with their clients (Owen, 2013). Given the historically understudied nature of CNM people and their experiences, it is conceivable that many clinicians would be less culturally comfortable addressing CNM paradigms openly with clients, especially if they are considering culture from a more restricted lens (e.g., race, ethnicity, sexual orientation). This may lead to missed cultural opportunities and greater hesitation to discuss content areas salient to the client. To enhance cultural comfort with CNM topics, clinicians might consider becoming more familiar with clinical and/or research literature involving these populations. They can become more familiar with the role of phenomena that manifest in these clients, such as the term “compersion” and its role and juxtaposition with jealousy in CNM relationships. Additional actions to facilitate cultural comfort may involve deliberate practice exercises (see Rousmaniere, 2016) in the exploration of CNM content, as well as discussing with supervisors and/or peer consultation groups topics that may be particularly salient to CNM populations. These suggestions are non-exhaustive, and the authors encourage clinicians to be creative in their exploration of strategies to facilitate cultural comfort so they will be less nervous or anxious to discuss these topics in session with clients.

#### Tentative framework for clinical work with jealousy

Finally, when dealing with jealousy in clients of all relationship structures, clinicians might consider the exploration of the jealousy strategies employed successfully by the CNM participants in this study. Though no strategic framework was specifically sought by the researchers, analysis of the transcripts revealed commonalities among the CNM participants’ stories of successful management of jealousy. These themes can be described with a 5-step framework or “roadmap” through jealousy created by the first author: notice, name, narrate, navigate needs, and nurture compersion. The steps in this roadmap could potentially be used during treatment planning and adapted to suit the clinician’s preferred modality. Each step will be described briefly here.

Step 1. Notice: CNM participants consistently reported that if they noticed jealousy early it was easier to manage. Noticing took the form of attending to sensations in the body, racing or uncomfortable thoughts, and non-specific feelings of insecurity or threat and labeling them as clues that jealousy may be present. Some individuals may struggle to identify bodily sensations of tender emotional states, as did more than half the participants in the monogamous group, which indicates an opportunity for clinicians to include somatic or embodied awareness practices during treatment planning for jealousy.

Step 2. Name: Naming that jealousy is present was a key move for those participants who described jealousy as a manageable emotion. Conversely, several participants across groups described a specific discomfort when naming their jealous feelings because they experienced internalized shame and/or judgment from their partners. The five CNM participants who had destigmatized jealousy were able to work effectively to not only manage the emotional content but address unmet relational needs. They also noted a range of other feelings that appeared to be connected to their jealousy, including anger, sadness, fear, shame, envy, and resentment. Clinicians could assist clients in teasing apart their jealousy, naming the constituent emotions, and work with them to apply emotional regulation strategies relevant to their chosen therapeutic modality to ameliorate the intensity of jealousy.

Step 3. Narrate: Clinicians might consider utilizing narrative techniques to unpack the stories from which their clients are building a personal meaning of jealousy. All six of the CNM participants had grown more comfortable with jealousy through a prolonged process of re-imagining jealousy as a source of information without automatically presuming their relationship was in imminent danger. Several also noted that they actively critiqued the common cultural narratives of jealousy disseminated through literature, films, television, and social media content. Narrative approaches may be appropriate for clients, particularly those who tend to engage in hyperbolic thought habits or who have entrenched ideas about the meaning of jealousy that are not serving their relational goals (Trzebiński et al., 2021).

Step 4. Navigate needs: The results of this study suggest that the CNM participants used some common skills to respond to the feelings of jealousy: explicit communication, boundary-setting, agreement making, emotional attunement, and rupture and repair skills. The effective use of these skills by the CNM participants suggests that psychoeducational support in developing and implementing relational skills may be a useful clinical strategy. However, successful application of these skills requires an understanding of one’s needs and how one wants them to be met. Therefore, it may be advantageous for clinicians to support clients in identifying and responding to their personal and relational needs in addition to learning communication strategies.

Step 5. Nurture compersion: As described above, compersion is the emotional state of feeling positively about one’s partner’s joyful experiences with other people (Brunning, 2020). Compersion was not felt strongly by all CNM participants, nor did they report a consistent experience of compersion for their partners’ pleasurable connections. Yet, four of the five CNM participants did feel compersion both as a spontaneous affective state, and as an attitude they consciously nurtured in order to counterbalance feelings of jealousy. Research indicates that compersion may be experienced as either an attitudinal state or an embodied one, suggesting that differences in compersive feelings are typical and could be developed over time with intentional practice (Thouin-Savard, 2021). Compersion may not be easily accessed by some individuals, including some clinicians, which could hinder the imagination that positive feelings of sympathetic joy could exist at the same time as jealousy, yet CNM participants noted that they had experienced this paradox personally (Mogilski et al., 2019).

This tentative five-step “jealousy roadmap” is offered in the spirit of clinical creativity. The success pathway that emerged from the CNM participants’ narratives was unexpected. The commonalities among their stories made it clear that taking a proactive approach to jealousy management was not only possible but, in these limited cases at least, advantageous. This roadmap could be adapted to suit many modalities of clinical practice including CBT, dialectical behavior therapy, narrative, psychodynamic, and somatic approaches. The adaptations might include reorganization of the steps or the repurposing of specific strategies already established during earlier therapeutic sessions with a client. For example, noticing sensations or identifying one’s needs may play an integral part of many interventions, allowing the clinician to work with jealousy more expediently when it arrives in session.

### Limitations and future directions

Several limitations characterize this study. First, it is important to consider the potential impact of self-selection bias in the present sample. Given the sensitive nature of jealousy in romantic relationships, the individuals who agreed to participate may have had unique characteristics, perspectives, or experiences compared to those who did not participate. For example, those willing to discuss their experiences of romantic jealousy may have been particularly self-aware, may have been more open to discussing what is often described as a difficult emotion, and/or discussed this topic more openly with partners compared with those individuals who did not participate. Second, while the homogeneous focus in this study on cis-women was consistent with the parameters of IPA, it limits the generalizability of the study findings to other gender identities. In the same way some experiences of jealousy differed between the monogamous and CNM samples, it is likely the experiences of jealousy vary between genders (e.g., cis-man, cis-woman, trans, non-binary). Third, individuals who identify as CNM are far from monolithic. The highly varied ways CNM individuals can structure their relationships may have impacted the homogeneity of the CNM group, though they all utilized the identity label CNM. Lastly, the cultural context of these participants is important to consider, especially when considering the role of jealousy in monogamy vs. CNM. Culture may play a role at the macro level (e.g., what are the laws, expectations, and norms of an individual’s home country), micro level (e.g., what is deemed permissible in an individual’s family of origin), and anywhere in between (e.g., is the individual in a location with other CNM people and support systems). The cultural context of the participants undoubtedly shaped this study’s findings.

Limitations aside, in this qualitative comparison of jealousy, participants were readily able to identify some clear experiences of jealousy and strategies for its management. Given the role of jealousy in romantic relationships, and its varied presentations between monogamous and CNM individuals, it is important for researchers and clinicians to continue addressing experiences of jealousy. Future qualitative work might examine different experiences of jealousy across demographic lines (e.g., experiences of jealousy in cis-men or trans people), while future quantitative work might systematically examine the norms and strategies identified in the present study on a larger scale and explore more complex models that may emerge beyond the descriptive nature of the themes established in this study. Additionally, clinicians are encouraged to more mindfully address jealousy as outlined in the discussion above. Empirical and clinical work in these areas, especially in CNM populations, remains in its infancy and requires additional attention if the field is to adequately meet the needs of these communities.

## Appendix A

### Interview Schedule

Part I: Preliminary Questions [SPEND LESS TIME ON THESE RELATIVE TO THE REMAINDER OF THE INTERVIEW]

- What made you want to participate in this interview?
- Would you describe what your home/romantic life looks like?
  - Who do you live with?
  - How do you spend your day?
  - Who do you share a romantic connection with?

Part II: Exploratory Questions

- Would you please define jealousy to me in your own words?
- How have you experienced jealousy in the past?
- How are you currently experiencing jealousy?

***Optional encouragement: “Please think carefully about this and describe as fully and vividly as possible. We really want to get a clear sense of the way jealousy feels for you”.

- How did you identify that you were feeling jealous?
- What sensations do you experience in your body when you are jealous?
- What type of situation is most likely to inspire jealousy in you?
- What do you do when you feel jealous?
- What other emotions come up when you feel jealous?
- What types of people have inspired jealousy in your life?
- Have you ever been the object of someone else’s jealousy?
- Are there any activities or situations you avoid in order to avoid jealousy?
- Would you say that you are more or less jealous than other people in general?
- What role, if any, should jealousy play in a romantic relationship?
- What do you do, if anything, to protect yourself from or prevent jealousy?
- Have you ever experienced intense or violent behavior that was connected to jealousy?

Part III: Psychodynamic Explorations

- What was your childhood like- siblings? sibling rivalry? parental attempts at equity?
- What did you learn from your caregivers or teachers about jealousy?
- How do you express jealousy? Nonverbally? Verbally? Emotively? Artistically?
- Have you ever written any poetry, fiction, or made art or music related to feeling jealous?
- If so, would you be interested in sharing that as part of this data collection?
- Have you ever had any dreams about jealousy?
  - If so, would you describe one or more?

Part IV: Wrap-up Questions

As we wrap up our interview, I want to thank you for sharing your experiences with me. A predesigned set of questions may not have gotten into everything you want to share.

- Is there anything you would like to add about your experiences of jealousy?
- What was it like to reflect on jealousy during this interview?
